# The combined impact of inflammation and oxidative balance on mortality risk in rheumatoid arthritis patients: A retrospective cohort study

**DOI:** 10.1097/MD.0000000000048043

**Published:** 2026-03-27

**Authors:** Zhong Wen, Shunbing Wang, Yuzhi Li, Xin Tu, Quanbo Zhang, Yufeng Qing

**Affiliations:** a Department of Rheumatology and Immunology, Affiliated Hospital of North Sichuan Medical College, Nanchong, Sichuan, China; b School of Clinical Medicine, North Sichuan Medical College, Nanchong, Sichuan, China; c Research Center of Hyperuricemia and Gout, Affiliated Hospital of North Sichuan Medical College, Nanchong, Sichuan, China; d Department of Geriatrics, Affiliated Hospital of North Sichuan Medical College, Nanchong, Sichuan, China.

**Keywords:** mortality, NHANES, oxidative balance score, rheumatoid arthritis, systemic inflammation response index

## Abstract

Oxidative stress and systemic inflammation play pivotal roles in rheumatoid arthritis (RA) progression. However, the combined impact of oxidative balance and inflammatory status on mortality risk in RA patients remains insufficiently investigated. This study aimed to explore these potential associations. We analyzed data from 24,552 participants in the National Health and Nutrition Examination Survey (NHANES, 2007–2018). Oxidative balance was assessed using the oxidative balance score (OBS), while inflammatory status was evaluated via the systemic inflammation response index (SIRI). Cox proportional-hazards models, Kaplan–Meier survival curves, and mediation analysis were employed to examine independent and joint associations. After adjusting for confounders, each 1-point increase in the OBS was associated with a 4 % reduction in mortality (HR = 0.96, 95 % CI 0.92–1.00, *P* = .047), and participants in the high OBS group exhibited a 37 % lower mortality risk than those with low OBS (HR = 0.63, 95 % CI 0.42–0.94, *P* = .023). Whereas every 1-unit rise in the SIRI elevated the risk by 14 % (HR = 1.14, 95 % CI 1.04–1.25, *P* = .005), and individuals in the high SIRI group faced more than double the risk seen in their low SIRI counterparts (HR = 2.30, 95 % CI 1.18–4.49, *P* = .014). A potential protective effect of an antioxidant-oriented diet and lifestyle was suggested only in patients with systemic inflammation, although the association did not reach conventional statistical significance (HR = 0.59, 95% CI 0.34–1.01, *P* = .053). No significant benefit was observed among patients without systemic inflammation. Spearman correlation revealed a weak inverse association between OBS and SIRI (R = -0.095, *P* = .007). Mediation analysis confirmed that antioxidants attenuate mortality risk in RA patients by reducing inflammatory response, with a mediation proportion of 14.6% (*P* <.001). Antioxidants exhibit protective effects against inflammation-related mortality in RA patients, suggesting that enhancing oxidative balance may serve as a potential intervention strategy to prevent RA associated deaths.

## 1. Introduction

Rheumatoid arthritis (RA), a prevalent chronic autoimmune disorder, is characterized by persistent joint inflammation and structural damage.^[1]^ The pathogenesis of RA involves complex immunological dysregulation, including the activation of autoreactive T and B cells, the production of pro-inflammatorycytokines, and the formation of autoantibodies, which collectively drive synovitis and systemic complications.^[2]^ Recent advances integrating multi-omics analyses further highlight the interplay between immune cell infiltration, novel regulatory cell death pathways (e.g., ferroptosis), and RA pathology.^[3]^ The 2017 Global Burden of Disease study reported a 7.4% increase in the age-standardized global prevalence of RA and an 8.2% increase in its incidence from 1990 to 2017.^[4]^ A large cohort study demonstrated that individuals with RA have a 54% higher risk of mortality,^[5]^ compared with the general population. Although extensive studies had been conducted on its etiology and treatment, patient prognosis remains influenced by multiple factors. Among these, inflammatory processes and oxidative stress have been identified as key biomarkers that play pivotal roles in disease initiation and progression.

In recent years, investigations into oxidative stress have gained increasing attention. The oxidative balance score (OBS), an indicator assessing the balance between oxidative and antioxidative states in vivo, not only reflects the body’s antioxidant capacity but may also influence chronic disease outcomes.^[6]^ Existing studies have demonstrated that lower antioxidant capacity is associated with increased disease activity and joint damage in RA patients,^[7]^ while a higher OBS correlates with reduced RA incidence risk.^[8]^ Conversely, the systemic inflammation response index (SIRI), widely employed to evaluate systemic inflammatory status, has shown growing associations with both RA development risk and mortality. A series of prospective and retrospective studies have demonstrated that elevated inflammatory levels are consistently associated with increased mortality risk in RA patients.^[9,10]^ Nevertheless, the interplay between inflammatory responses and oxidative balance, along with their collective impact on RA prognosis, requires further elucidation.

Our investigation will utilize the National Health and Nutrition Examination Survey (NHANES) database to examine oxidative balance and inflammation in both RA and non-RA populations, thereby exploring their relationship with RA patient mortality risk. The objective of this study is to investigate the independent and combined effects of oxidative balance and inflammation on long-term prognosis in RA patients, providing novel insights for prediction and intervention in RA survivors.

## 2. Materials and methods

This retrospective cohort study employed data from the NHANES to systematically evaluate the relationship between OBS, SIRI, and mortality risk in patients with RA. NHANES represents a nationally representative, continuous surveillance program that comprehensively assesses the health and nutritional status of the civilian, noninstitutionalized US population. The study protocol received full ethical approval from the National Center for Health Statistics Institutional Review Board, with documented written informed consent obtained from all participating individuals prior to their inclusion in the survey.

**Figure 1.**
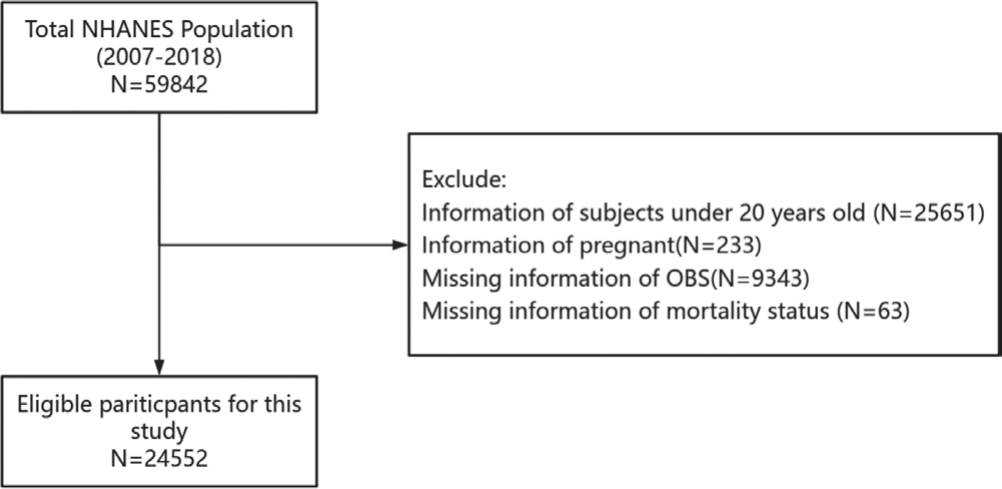
Screening process for participants.

The analysis comprised 24,552 participants enrolled across 5 biennial survey cycles (2007–2018). Within this population, 812 individuals were identified with physician-diagnosed RA, while the remaining participants constituted the non-RA comparison group. Inclusion criteria mandated: age ≥20 years; self-reported diagnosis of RA. All study subjects completed standardized health questionnaires, underwent comprehensive physical measurements, and provided biological specimens for laboratory analyses that included all essential biomarker determinations. Exclusion criteria were rigorously applied to eliminate: pregnant individuals; participants with incomplete OBS data; and cases with indeterminate mortality status. The complete participant selection algorithm is presented in Figure 1.

The OBS was calculated using 24-hour dietary recall data collected through NHANES protocols, incorporating quantitative assessments of 16 distinct dietary nutrients and 4 lifestyle factors.^[11]^ The scoring algorithm assigned positive values to antioxidant nutrients (including vitamin C, vitamin E, β-carotene, and polyphenols) based on intake quartiles, with progressively higher scores allocated to greater intake levels. Conversely, pro-oxidant substances (such as saturated fats and high-glycemic-index foods) received negative scoring when consumption exceeded predetermined threshold values. The final OBS was derived by summing all positive antioxidant scores and subtracting the cumulative negative pro-oxidant scores. The negative values indicated a propensity for oxidative stress, whereas positive values denoted antioxidant predominance, with higher aggregate scores reflecting superior antioxidant capacity. The SIRI was computed using the following formula: SIRI = (neutrophil count × monocyte count)/ lymphocyte count. The complete blood count data were obtained from the NHANES laboratory component. Blood collection followed standardized protocols during Mobile Examination Center visits. To account for variability in collection time, we applied NHANES fasting weights to adjust for collection time variations.

The mortality status and cause-of-death data were obtained through probabilistic linkage with the national death index maintained by the Centers for Disease Control and Prevention (CDC). The follow-up interval extended from each participant’s baseline examination date until either the documented date of death, or December 31, 2019.

The covariates considered in this study included demographic variables (age, sex, race/ethnicity, education attainment, marital status, and family income) and health-related factors (smoking, alcohol consumption, physical activity, hypertension, diabetes, and cardiovascular disease history).

All analyses incorporated sampling weights, clustering, and stratification to ensure nationally representative estimates. Continuous variables were expressed as mean ± SD, while categorical variables were presented as frequencies and percentages. Differences between RA and non-RA participants were assessed using independent *t*-tests for continuous variables and chi-square tests for categorical variables. To explore the relationship between oxidative balance and systemic inflammation, Spearman correlation analysis was conducted between OBS and SIRI. Restricted cubic spline (RCS) analysis assessed nonlinear associations between OBS, SIRI, and mortality. Kaplan–Meier (KM) survival analysis illustrated survival differences across groups, with log-rank tests for statistical comparison. The OBS and SIRI were categorized into 4 groups (low/high OBS groups and low/high SIRI groups) based on RCS thresholds. Cox proportional-hazards regression models were employed to evaluate mortality risks across these groups, with follow-up duration calculated from study entry until death or the last recorded contact. The model 1 (unadjusted) included only OBS and SIRI; Model 2 additionally accounted for age, and sex; and Model 3 further adjusted for BMI, race, education attainment, marital status, and family income, alcohol consumption, smoking, physical activity, hypertension, diabetes, cancer, Osteoporosis, and cardiovascular disease history. To examine the combined effects of OBS and SIRI on mortality risks, subgroup analyses were conducted using RCS thresholds. Mediation analysis with 5000 bootstrap resamples was performed to assess whether antioxidant status influenced mortality risk through its effect on systemic inflammation. Mediation analysis with 5000 bootstrap resamples was performed to assess whether antioxidant status influenced mortality risk through its effect on systemic inflammation. Variables with missing data were imputed using multiple imputation methods. Finally, sensitivity analyses were conducted by excluding participants aged under 45 years to validate the robustness of our models. All analyses were performed using R software (version 4.3.1), with statistical significance set at *P* <.05.

## 3. Results

### 3.1. Participant baseline characteristics

Among the 24,552 participants included in this study, 812 (3.3%) were diagnosed with RA. Baseline characteristics are presented in Table 1. Compared to non-RA individuals, RA patients were significantly older (mean age 58 ± 13 vs 49 ± 17 years, *P* <.001) and more likely to be female (59% vs 52%, *P* = .028). RA patients exhibited a higher prevalence of smoking, hypertension, and diabetes compared to their non-RA counterparts (*P* <.05 for all). Furthermore, the prevalence of key comorbidities, including a history of cancer (17% vs 11%, *P* <.001) and osteoporosis (8.1% vs 2.0%, *P* <.001), was significantly higher in the RA group. In contrast, non-RA participants demonstrated healthier lifestyle patterns overall. Regarding inflammatory and oxidative status, RA patients demonstrated significantly lower OBS (19 ± 7 vs 21 ± 7, *P* <.001), indicating diminished antioxidant capacity. Although SIRI levels were nominally higher in RA patients (1.44 ± 1.26 vs 1.26 ± 0.86), this difference did not achieve statistical significance (*P* = .14).

**Table 1:** Baseline characteristics of NHANES individuals.

Characteristic	Overall N = 24552 (100%)	Non-RA N = 23740 (96.7%)	RA N = 812 (3.3%)	*P*-value
Age (yr)	49 ± 17	49 ± 17	58 ± 13	<.001
Sex				
Female	12,724 (52%)	12,247 (52%)	477 (59%)	.028
Male	11,828 (48%)	11,493 (48%)	335 (41%)	
Race				
Mexican American	3593 (8.1%)	3481 (8.1%)	112 (7.9%)	.025
Other Hispanic	2543 (5.6%)	2460 (5.6%)	83 (5.8%)	
Non-Hispanic White	10,717 (68%)	10,421 (68%)	296 (64%)	
Non-Hispanic Black	5058 (11%)	4804 (10%)	254 (15%)	
Other/multiracial	2641 (7.4%)	2574 (7.4%)	67 (6.7%)	
Marital status				
Married	13,002 (56%)	12,598 (56%)	404 (56%)	<.001
Never married	4086 (17%)	4014 (17%)	72 (7.6%)	
Living with partner	1939 (7.8%)	1890 (7.8%)	49 (6.6%)	
other	5525 (19%)	5238 (19%)	287 (29%)	
Education attainment				
High school or less	11,177 (38%)	10,754 (37%)	423 (46%)	<.001
Some College	7305 (31%)	7026 (31%)	279 (39%)	
College Graduate or above	6070 (31%)	5960 (31%)	110 (15%)	
Family income				
High income	4609 (28%)	4502 (28%)	107 (21%)	<.001
Low income	8471 (25%)	8108 (24%)	363 (35%)	
Medium income	11,472 (48%)	11,130 (48%)	342 (44%)	
Alcohol consumption				
nondrinker	6977 (22%)	6693 (22%)	284 (29%)	.002
1–5 drinks/mo	12,221 (50%)	11,816 (50%)	405 (54%)	
5–10 drinks/mo	1888 (9.4%)	1840 (9.5%)	48 (5.6%)	
10 + drinks/mo	3466 (18%)	3391 (18%)	75 (12%)	
Smoke group				
Never smoker	13,618 (55%)	13,230 (56%)	388 (45%)	<.001
Former smoker	6162 (26%)	5919 (26%)	243 (31%)	
Current smoker	4772 (19%)	4591 (19%)	181 (25%)	
BMI group				
Normal (18.5 to < 25)	6317 (27%)	6176 (27%)	141 (22%)	.026
Obese (30 or greater)	9766 (39%)	9364 (38%)	402 (46%)	
Overweight (25 to < 30)	8129 (33%)	7869 (33%)	260 (31%)	
Underweight (<18.5)	340 (1.3%)	331 (1.3%)	9 (1.2%)	
Physical activity group				
Sufficient	14,419 (63%)	14,004 (64%)	415 (53%)	<.001
Insufficient	3168 (13%)	3077 (13%)	91 (12%)	
Low	530 (1.9%)	512 (1.9%)	18 (2.1%)	
Inactive	6435 (22%)	6147 (21%)	288 (32%)	
Hypertension				
Yes	10,984 (40%)	10,425 (39%)	559 (62%)	<.001
No	13,568 (60%)	13,315 (61%)	253 (38%)	
Diabetes				
Yes	4494 (14%)	4236 (13%)	258 (25%)	<.001
No	20,058 (86%)	19,504 (87%)	554 (75%)	
Cancer				
Yes	2486 (11%)	2358 (11%)	128 (17%)	<.001
No	22,066 (89%)	21,382 (89%)	684 (83%)	
Osteoporosis				
Yes	515 (2.2%)	457 (2.0%)	58 (8.1%)	<.001
No	4452 (18%)	4146 (18%)	306 (37%)	
Unknown	19,585 (79%)	19,137 (80%)	448 (55%)	
OBS dietary	17 ± 7	17 ± 7	16 ± 7	.003
OBS lifestyle	4.19 ± 1.57	4.20 ± 1.57	3.77 ± 1.54	<.001
OBS	21 ± 7	21 ± 7	19 ± 7	<.001
SIRI	1.26 ± 0.87	1.26 ± 0.86	1.44 ± 1.26	.14

Median (IQR) for continuous; sample size (unweighted n) and weighted percentage (%); design-based Kruskal–Wallis test; Pearson X^2^: Rao and Scott adjustment; *P* < .05 was considered significant.

BMI = body mass index, OBS = oxidative balance score, RA = rheumatoid arthritis, SIRI = systemic inflammation response index.

### 3.2. RCS and KM survival analyses

**Figure 2.**
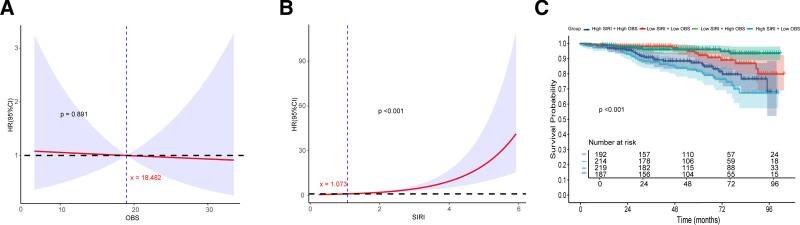
RCS Analysis and KM survival analysis between OBS, SIRI and Mortality of RA. (A and B): RCS curves of OBS and SIRI related to mortality of RA. (C) KM survival analysis based on OBS and SIRI groups. KM = Kaplan Meier, OBS = oxidative balance score, RA = rheumatoid arthritis = RCS = restricted cubic splines, SIRI = systemic inflammation response index.

The RCS analysis was performed to explore the relationship between SIRI, OBS and mortality of RA. The results revealed a significant nonlinear association between SIRI and mortality risk (*P* < .001), with the hazard ratio exceeding 1 as SIRI increased above 1.073, indicating higher risk of adverse outcomes. In contrast, the nonlinear relationship between OBS and RA mortality was not statistically significant (*P* = .891). The KM survival analysis was performed based on the combinations of SIRI and OBS groups. The log-rank test indicated statistically significant differences among the 4 groups (*P* < .0001). Notably, individuals with high SIRI and low OBS exhibited the lowest survival probability, while those with low SIRI and high OBS had the most favorable survival outcomes (Fig. 2).

### 3.3. Cox regression analysis

As shown in Table 2, the weighted Cox proportional-hazards analysis revealed opposing effects of oxidative balance and systemic inflammation on mortality risk of RA. When treated as continuous variables, each 1-point increase in the OBS was associated with a 4 % reduction in mortality (HR = 0.96, 95 % CI 0.92–1.00, *P* = .047), whereas every 1-unit rise in the SIRI elevated the risk by 14 % (HR = 1.14, 95 % CI 1.04–1.25, *P* = .005). After dichotomization, participants in the high OBS group exhibited a 37 % lower mortality risk than those with low OBS (HR = 0.63, 95 % CI 0.42–0.94, *P* = .023), while individuals in the high SIRI group faced more than double the risk seen in their low SIRI counterparts (HR = 2.30, 95 % CI 1.18–4.49, *P* = .014).

**Table 2:** Association of OBS and SIRI with mortality in RA patients.

Characteristic	Model 1		Model 2		Model 3	
	95% CI	*P*	95% CI	*P*	95% CI	*P*
Continuous variable						
OBS	0.95 (0.92–1.00)	.014	0.96 (0.92–1.00)	.039	0.96 (0.92–1.00)	.047
SIRI	1.21 (1.14–1.29)	<.001	1.13 (1.06–1.21)	<.001	1.14 (1.04–1.25)	.005
Categorical variable						
OBS group	–	–	–	–	–	–
Low OBS	–	–	–	–	–	–
High OBS	0.51 (0.30–0.87)	.014	0.61 (0.38–0.99)	.045	0.63 (0.42–0.94)	.023
SIRI group						
Low SIRI	–	–	–	–	–	–
High SIRI	3.94 (2.23–6.98)	<.001	2.56 (1.38–4.75)	.003	2.30 (1.18–4.49)	.014

Model 1: included only OBS and SIRI; Model 2: additionally accounted for age, sex; Model 3: further adjusted for BMI, race, education attainment, marital status, and family income, alcohol consumption, smoking, physical activity, hypertension, diabetes, cancer, osteoporosis and cardiovascular disease history.

BMI = body mass index, OBS = oxidative balance score, RA = rheumatoid arthritis, SIRI = systemic inflammation response index.

Further insights were obtained from stratified analyses by systemic inflammation status (Table 3). Among participants with elevated SIRI levels, a high OBS exhibited a borderline significant association with reduced mortality (HR = 0.59, 95% CI 0.34–1.01, *P* = .053). By contrast, no statistically significant association between OBS and mortality was detected in the low SIRI subgroup (HR = 0.41, 95% CI 0.11–1.56, *P* = .200). These results indicate that the potential survival benefit associated with a high antioxidant balance score appears predominantly among individuals with increased systemic inflammation.

**Table 3:** The stratified analysis of OBS and SIRI associations with mortality in RA patients by SIRI levels.

Characteristic	Low SIRI group		High SIRI group	
	95% CI	*P*	95% CI	*P*
Model 1				
OBS group	–	–	–	–
Low OBS	–	–	–	–
High OBS	0.44 (0.14–1.33)	.140	0.63 (0.34–0.99)	.045
Model 2				
OBS group	–	–	–	–
Low OBS	–	–	–	–
High OBS	0.56 (0.18–1.77)	.300	0.62 (0.36–1.09)	.010
Model 3				
OBS group	–	–	–	–
Low OBS	–	–	–	–
High OBS	0.41 (0.11–1.56)	.200	0.59 (0.34–1.01)	.053

Model 1: included only OBS; Model 2: additionally accounted for age, sex; Model 3: further adjusted for BMI, race, education attainment, marital status, and family income, alcohol consumption, smoking, physical activity, hypertension, diabetes, cancer, osteoporosis and cardiovascular disease history.

OBS = oxidative balance score, RA = rheumatoid arthritis, SIRI = systemic inflammation response index.

### 3.4. Association and mediation analysis

Spearman correlation revealed a weak inverse association between OBS and SIRI (R = -0.095, *P* = .007). Mediation analysis revealed that the antioxidant response (reflected by OBS) partially mediated the effect of systemic inflammation levels (indicated by SIRI) on RA mortality, with a mediation proportion of 14.6% (*P* <.001) (Table 4). This supports the pathway that antioxidant status influences risk partly by attenuating systemic inflammation.

**Table 4:** Mediation analysis of the impact of OBS on the relationship between SIRI and mortality.

Effect decomposition	Coefficient (95% CI)	*P*-value	Proportion mediated, %
Indirect effect	−0.00063 (−0.00255 to 0.00)	<.001	14.61
Direct effect	−0.00370 (−0.00755 to 0.00)	.04	85.39
Total effect	−0.00433 (−0.00882 to 0.00)	<.001	100

OBS = oxidative balance score, SIRI = systemic inflammation response index.

### 3.5. Sensitivity analysis

To assess the robustness of the observed associations, a sensitivity analysis was conducted by excluding participants under 45 years of age. As shown in Table 5, after full adjustment in Model 3, the high OBS group maintained a significant protective association with mortality (HR 0.65, 95% CI 0.44–0.97, *P* = .037). Conversely, the high SIRI group demonstrated a 2.15-fold increased mortality risk (95% CI 1.12–4.15, *P* = .022).

**Table 5:** Sensitivity analysis of OBS and SIRI associations with mortality in RA patients.

Characteristic	Model 1		Model 2		Model 3	
	95% CI	*P*	95% CI	*P*	95% CI	*P*
OBS group	–	–	–	–	–	–
Low OBS	–		–		–	
High OBS	0.55 (0.33–1.00)	.021	0.65 (0.41–1.03)	.067	0.65 (0.44–0.97)	.037
SIRI group	–	–	–	–	–	–
Low SIRI	–	–	–	–	–	–
High SIRI	3.81 (2.20–6.60)	<.001	2.44 (1.34–4.43)	.003	2.15 (1.12–4.15)	.022

Model 1: included only OBS and SIRI; Model 2: additionally accounted for age, sex; Model 3: further adjusted for BMI, race, education attainment, marital status, and family income, alcohol consumption, smoking, physical activity, hypertension, diabetes, cancer, osteoporosis and cardiovascular disease history.

OBS = oxidative balance score, RA = rheumatoid arthritis, SIRI = systemic inflammation response index.

## 4. Discussion

Our study provided novel insights into the relationship between oxidative balance, systemic inflammation, and mortality risk in patients with RA using data from the NHANES database. The findings demonstrated that a lower OBS is significantly associated with an elevated risk of mortality, while heightened systemic inflammation, as reflected by the SIRI, further exacerbates this risk. Importantly, our mediation analysis suggested that antioxidant capacity partially attenuated the detrimental effects of inflammation on mortality risk in RA patients.

The OBS is a comprehensive indicator that evaluates the body’s antioxidant and oxidative status, determined by multiple factors including dietary intake of antioxidants, exposure to oxidative stress, and the activity of endogenous antioxidants.^[6]^ The calculation of OBS typically incorporates well-known antioxidant nutrients such as vitamins C and E, β-carotene, selenium, as well as certain phytonutrients (e.g., polyphenolic compounds).^[12]^ The intake levels of these elements directly influence the body’s oxidative stress status, thereby affecting overall health. Existing studies have demonstrated a close association between oxidative stress and various chronic inflammatory diseases, particularly in RA and other autoimmune disorders.^[13,14]^ Zhang et al reported that a higher OBS was significantly associated with a reduced risk of RA incidence and this protective association was more pronounced in patients with preexisting chronic kidney disease and cardiovascular diseases.^[8]^ Additionally, Veselinovic et al supported a significant positive correlation between oxidative/nitrosative stress levels and disease severity in RA patients.^[15]^ In our study, RA patients exhibited significantly lower OBS values compared to non-RA individuals (RA: 19 ± 7 vs non-RA: 21 ± 7, *P* <.001), which aligns with the aforementioned literature. The novelty of our study lies in specifically evaluating the direct impact of OBS on mortality risk. We found that each 1-point increase in the OBS was associated with a 4 % reduction in mortality (HR = 0.96, 95 % CI 0.92–1.00, *P* = .047), and participants in the high OBS group exhibited a 37 % lower mortality risk than those with low OBS (HR = 0.63, 95 % CI 0.42–0.94, *P* = .023), suggesting that improving oxidative status may hold significant value for enhancing survival outcomes in RA patients.

The SIRI, a recently proposed inflammatory biomarker derived from peripheral blood neutrophil, monocyte, and lymphocyte counts, has emerged as a reliable indicator of systemic inflammatory status. Its clinical utility has been increasingly recognized across various pathological conditions, particularly in stroke,^[16]^ cognitive performance,^[17]^ infectious diseases,^[18]^ malignancies,^[19]^ and autoimmune disorders such as RA.^[20]^ Accumulating evidence has established SIRI as a robust prognostic marker in RA. Wang et al identified a significant nonlinear positive correlation between SIRI and both mortality and cardiovascular mortality in patients with RA.^[21]^ Furthermore, González-Sierra et al demonstrated that elevated SIRI levels in RA patients were independently associated with increased risks of adverse cardiovascular events.^[22]^ Our findings further corroborate this perspective, demonstrating that every 1-unit rise in the SIRI elevated the risk by 14 % (HR = 1.14, 95 % CI 1.04–1.25, *P* = .005), and individuals in the high SIRI group faced more than double the risk seen in their low SIRI counterparts (HR = 2.30, 95 % CI 1.18–4.49, *P* = .014). Subgroup analysis revealed that concurrent high SIRI and high OBS status was associated with a 41% mortality risk reduction (HR = 0.59, 95 % CI 0.34–1.01, *P* = .053). These results indicated that elevated OBS significantly mitigated mortality risk among RA patients with high systemic inflammation, suggesting that maintaining redox homeostasis may confer particularly beneficial effects on survival outcomes in populations with heightened inflammatory status. KM survival curve provided additional supportive evidence, found that the concurrent high SIRI and low OBS status exhibited the poorest survival probability and most unfavorable prognosis. Conversely, concurrent low SIRI and high OBS status demonstrated the most favorable survival outcomes and optimal prognosis. Our mediation analysis further confirmed that antioxidants can reduce mortality risk in RA patients by lowering inflammatory response, with a mediation proportion of 14.6% (*P* <.001). This result, together with the observed inverse correlation between OBS and SIRI (R = -0.095, *P* = .007) and the selective benefit of high OBS in the high-inflammation subgroup, provides epidemiological evidence supporting a key mechanistic pathway wherein oxidative stress influences RA prognosis, at least partially, by promoting systemic inflammation.^[23]^ This indicated that low antioxidant response and high systemic inflammation levels are independent risk factors for RA prognosis, and their coexistence may further worsen outcomes.

The observed protective association of a higher OBS, particularly in the context of elevated SIRI, may be underpinned by key molecular regulatory networks. One such axis involves the transcription factor nuclear factor-kappa B (NF-κB), which is a master regulator of pro-inflammatory cytokine production, and nuclear factor erythroid 2-related factor 2 (Nrf2), which is the primary regulator of cellular antioxidant responses. In RA, persistent oxidative stress can activate NF-κB, perpetuating inflammation and tissue damage.^[24]^ Conversely, dietary antioxidants activate the Nrf2 pathway, enhancing the expression of endogenous antioxidant enzymes.^[23,25]^ Activated Nrf2 can also suppress NF-κB signaling, thereby breaking the vicious cycle. Our findings show that a higher OBS correlates with a lower SIRI and reduces mortality risk in patients with high inflammation, which aligns with this model. These findings suggest that a robust antioxidant status may help to restore the Nrf2/NF-κB balance, thereby mitigating the systemic inflammatory burden and its sequelae. This mechanistic perspective, supported by prior experimental work in RA,^[15]^ provides a plausible biological framework for our epidemiological observations and highlights potential therapeutic targets.

The novelty of our study lies in not only investigating the significant impact of OBS and SIRI on RA patient mortality risk, but also elucidating their interaction and proposing the potential role of antioxidants in mitigating inflammation. While previous studies have explored the individual relationships of OBS and SIRI with RA, the simultaneous examination of these 2 biomarkers’ effects on RA prognosis and their combined impact remains scarcely documented in the literature. Subgroup analysis revealed that patients in the high OBS group exhibited significant survival advantages, suggesting that improving oxidative status may play a crucial role in reducing mortality risk among RA patients. This finding provided a novel perspective for effective clinical interventions. Furthermore, mediation analysis demonstrated that antioxidant measures not only reduce inflammatory levels but also exert significant protective effects on survival, offering new directions for future clinical research.

The interpretation of our findings is informed by the study’s clinical and methodological context. The SIRI values were derived from standardized NHANES laboratory data, with the application of fasting subsample weights to enhance comparability by accounting for variability in blood collection timing. Additionally, the higher prevalence of comorbidities such as cancer history and osteoporosis in the RA cohort suggests that the measured systemic inflammation reflects the integrated burden of RA activity and associated health conditions, which is representative of real-world patient populations. Our study provides preliminary risk thresholds (SIRI >1.073 and low OBS ≤ 18.482) that could aid in stratifying RA patients at diagnosis. Future research should focus on designing interventions, such as antioxidant-rich dietary plans, specifically for this high-risk subgroup to evaluate their impact on clinical outcomes.

This investigation has several methodological constraints. Foremost, the reliance solely on baseline measurements precluded serial assessment of participants’ antioxidant profiles and inflammatory dynamics during follow-up. Additionally, certain diagnostic and comorbidity data originated from self-reported sources, potentially introducing classification inaccuracies. Moreover, the osteoporosis variable exhibited a substantial missing rate in the RA group; despite employing multiple imputation to handle this, the possibility of residual bias cannot be fully excluded due to the assumption of data missing at random. Furthermore, the OBS was calculated using data from 24-hour dietary recalls, which are subject to recall bias and measurement error despite NHANES employing standardized protocols to enhance accuracy. Finally, while multiple covariates were adjusted for confounding mitigation, residual influences from unmeasured variables cannot be definitively excluded.

## 5. Conclusions

In conclusion, our comprehensive analysis of OBS and SIRI provided novel evidence for understanding survival outcomes in RA patients. These findings underscored the importance of monitoring and intervening in both antioxidant status and inflammatory levels in RA management. Future clinical trials should be conducted to validate the long-term prognostic benefits of interventions targeting oxidative balance improvement in RA patients.

## Author contributions

**Data curation:** Yuzhi Li, Xin Tu.

**Formal analysis:** Zhong Wen.

**Methodology:** Zhong Wen.

**Validation:** Shunbing Wang, Quanbo Zhang.

**Writing – original draft:** Zhong Wen, Shunbing Wang.

**Writing – review & editing:** Yufeng Qing.
